# Functional Sensing Interfaces of PEDOT:PSS Organic Electrochemical Transistors for Chemical and Biological Sensors: A Mini Review

**DOI:** 10.3390/s19020218

**Published:** 2019-01-09

**Authors:** Jianjun Liao, Hewei Si, Xidong Zhang, Shiwei Lin

**Affiliations:** 1 State Key Laboratory of Marine Resource Utilization in South China Sea, Hainan University, Haikou 570228, China; liaojianjun008@hainu.edu.cn (J.L.); 16080500210016@hainu.edu.cn (H.S.); 17080500210026@hainu.edu.cn (X.Z.); 2 College of Ecology and Environment, Hainan University, Haikou 570228, China; 3 College of Materials and Chemical Engineering, Hainan University, Haikou 570228, China

**Keywords:** organic electrochemical transistors, interface functionalization, chemical sensors, biosensors

## Abstract

Organic electrochemical transistors (OECTs) are promising devices for applications in in vitro and in vivo measurements. OECTs have two important sensing interfaces for signal monitoring: One is the gate electrode surface; the other is the channel surface. This mini review introduced the new developments in chemical and biological detection of the two sensing interfaces. Specific focus was given on the modification technological approaches of the gate or channel surface. In particular, some unique strategies and surface designs aiming to facilitate signal-transduction and amplification were discussed. Several perspectives and current challenges of OECTs development were also briefly summarized.

## 1. Introduction

An organic electrochemical transistor (OECT) is a three-terminal device in which a conducting polymer channel is deposited on the source and the drain, while the gate is separated from the channel by an electrolyte. The conducting polymer channel is usually made of poly(3,4-ethylenedioxythiophene) doped with poly(styrene sulfonate) (PEDOT:PSS), a benchmark material that is commercially available and stable in aqueous medium. Because PEDOT:PSS is in direct contact with an electrolyte, OECTs can sensitively convert (bio)chemical signals into electronic ones, which make them particularly suitable for chemical and biological detection. Moreover, OECT devices combine several attractive advantages, like ease of fabrication, compatibility with flexible substrates, low operating voltage (<1 V), signal amplification and high transconductance, thereby having broad applications in ions [1], glucose [2,3,4,5], bacteria [6], dopamine [7,8,9], DNA [10,11], lactate [12], cell activities [13,14,15], and electrophysiological signals [16,17]. Of particular interest is the factor that a reference electrode is not necessary when used, and this feature is very important for wearable and textile sensors [18,19,20].

In a typical OECT, analytes contained in the electrolyte can be selectively detected on the surface of either the channel or the gate electrode. Any (bio)chemical reaction occurring on the gate or channel surface may induce the change of interfacial potential, and affect the channel current and the sensor response. To realize the sensitive detection of targets, different modification methods to the gate or channel surface should be chosen, and great achievements have been obtained. This review will focus on the development of chemical and biological sensors based on OECTs with PEDOT:PSS as the channel, and highlights the work on the functionalization of the gate or channel surface, especially some unique strategies and surface designs aiming to facilitate signal-transduction and amplification. Finally, some perspectives for future research and development will be discussed.

## 2. Working Principle of an Organic Electrochemical Transistor (OECT) Device

**Figure 1:**
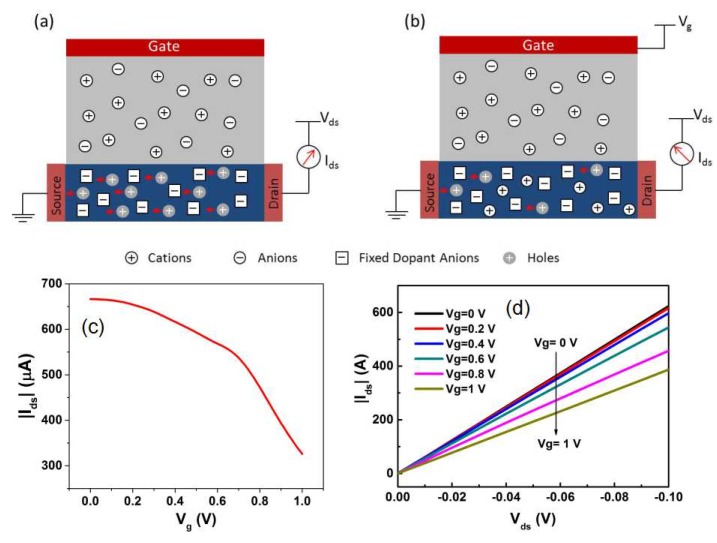
Schematic diagram of the working principle of an organic electrochemical transistor (OECT) device: (**a**) when no gate voltage is applied, poly(3,4-ethylenedioxythiophene) doped with poly(styrene sulfonate) (PEDOT:PSS) is conducting (ON state); (**b**) when a gate voltage is applied, the current is deceased due to the dedoping of the channel (OFF state). Typical transfer (**c**) and output (**d**) characteristics of an OECT measured in 0.1 M NaCl.

As shown in Figure 1a,b, the working principle is based on the doping/dedoping of the channel upon gate polarization [21,22,23]: at zero gate voltage, PEDOT:PSS is conducting (ON state). When a positive gate bias is applied, cations in the electrolyte would be injected into the PEDOT:PSS channel film and change its doping level, thereby decreasing the channel current (OFF state). In order to further clarify the mechanism, the transfer and output characteristics of an OECT measured in 0.1 M NaCl are given in Figure 1c,d. As seen, the drain-source current (Ids) decreases with the increasing gate voltage (V_g_), indicating that more cations are injected into the PEDOT:PSS film, and thus decreases the conductance of the organic semiconductor (PEDOT^+^:PSS^−^ + M^+^(aq) + e^−^ ↔ PEDOT^0^ + M^+^:PSS^−^).

Furthermore, a device model has been proposed by Bernards et al. [24,25], and the channel current of an OECT can be expressed by the following equation:

IDS=qμp0tWLVp(Vp−VGeff+VDS2)VDS, (when |VDS|<<|Vp−VGeff|)Vp=qp0tciVGeff=VG+Voffset

where *q* is the electronic charge, μ is the hole mobility, *P*_0_ is the initial hole density in the channel, *t* is the thickness of the organic semiconductor film, *W* is the channel width, *L* is the channel length, *V_p_* is the pinch-off voltage, *c_i_* is the effective gate capacitance per unit area of the transistor, *V*_G_^eff^ is the effective gate voltage, *V*_offset_ is the offset voltage related to the potential drop at the two interfaces: gate/electrolyte and electrolyte/channel. Therefore, OECTs are sensitive to the potential drop at the interface of electrolyte/channel or electrolyte/gate, and the change of potential drop at any one interface will modulate the *V*_G_^eff^ applied on the OECTs, thus changing the channel current.

**Figure 2:**
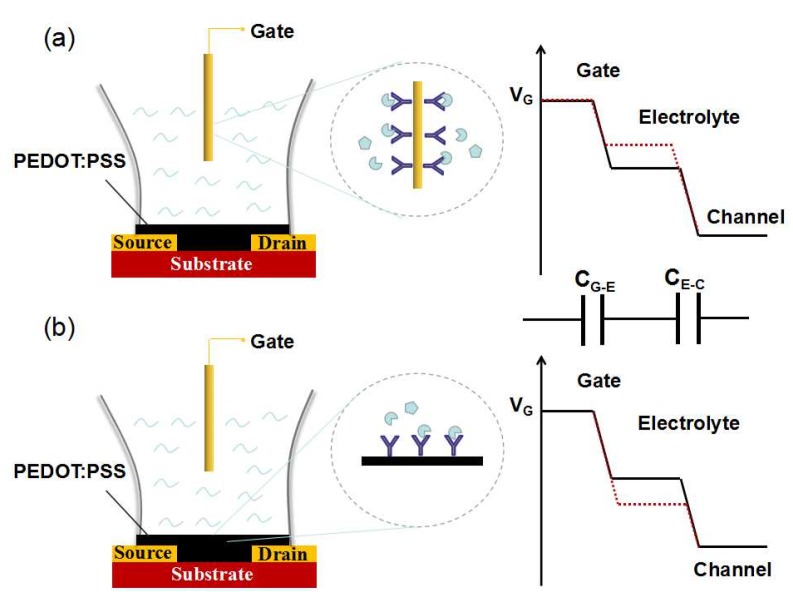
Schematic diagram of an OECT: (**a**) gate surface as the sensing interface, the potential distribution between gate and channel is changed by the reaction on the gate; (**b**) channel surface as the sensing interface, the potential distribution between the gate and channel is changed by the reaction on the channel.

According to the above analysis, as shown in Figure 2, OECTs have two important sensing interfaces for signal monitoring. One is the gate electrode surface, and the other is the channel surface. Since OECTs are sensitive to the change of potential, any minute potential changes on the gate or channel interface may lead to pronounced variation of channel current. Based on this, the design of the gate or channel interface is critical for the improvement of OECT performance.

## 3. Channel Surface as Sensing Interface

### 3.1. Geometry of Active Layer

Electrical readout of the electrophysiological signal has been of great interest in recent decades, but the bioelectric signal is usually weak and hard to be extracted from noise. Therefore, high transconductance (*g*_m_) and excellent signal to noise ratio (SNR) are two basic requirements for OECTs. Transconductance (*g*_m_ = ∆I_ds_/∆V_g_) refers to the signal amplification ability of OECTs [26,27]. High transconductance is usually related to high sensitivity of OECT-based sensors. Signal to noise ratio means the ratio of signal power to noise power contained in a recording. Usually, the detection limit, an important parameter of sensors, is calculated when SNR > 3 [28].

The maximum transconductance and signal to noise ratio are dependent on the thickness (d) and width/length (W/L) ratio of PEDOT:PSS channel. Liang et al. [17] studied the relationship between the channel layer thickness and transconductance. They found that the transconductance was dependent on the channel thickness when the W/L ratio was kept constantly. The thicker channel layer (115 nm) showed a higher transconductance (6 mS), while the channel layer with 50 nm thickness showed a low transconductance of 4 mS. The reason may be that the thicker channel film possesses a lower resistance, and under the same drain-source voltage, larger drain current will flow through the thicker channel. So higher transconductance (*g*_m_ = ∆I_ds_/∆V_g_) was obtained [29]. Because the size of a cell is small (in micro dimension) and the electrophysiological signals are weak, 27 pieces of OECTs were arranged into an array on a chip. The OECT array showed a high SNR of 7 dB and a high on-to-off ratio of 105. Furthermore, the OECT array was successfully used to monitor the action potential propagation of cells. Khodagholy et al. [26] found that the transconductance of an OECT with *L* = 10 μm, *W* = 10 μm was 4.0 mS, while the transconductance decreased to 2.7 mS when the channel length was decreased to *L* = 5 μm. They attributed the reason to the actual volume of the channel when the OECT operated. If the thickness of channel film remained the same, the shorter channel length can lead to faster response time and lower transconductance.

**Figure 3:**
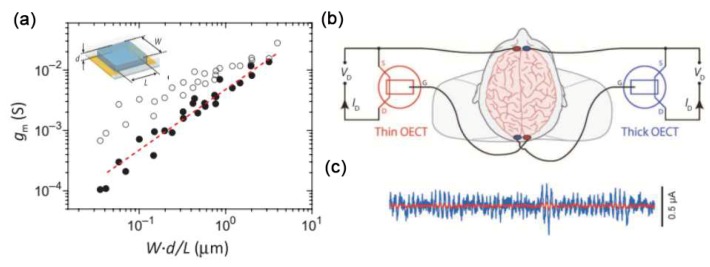
(**a**) The relationship between OECT transconductance (*g*_m_) and channel geometry (*W*·d/L). (**b**) Human electroencephalography recordings with thin OECT (red) and thick OECT (blue). (**c**) Electroencephalography signals recorded by the two OECTs, thin OECT (red) and thick OECT (blue). Figure reproduced with permission from reference [30].

As shown in Figure 3a, Rivnay et al. [30] systematically studied the geometry-performance relationship of OECTs and found that the transconductance was proportional to the channel volume *W*·d/L. In order to demonstrate the conclusion, they measured electroencephalography on a human volunteer using two different geometrical OECTs. As shown in Figure 3b, two OECT devices with the same channel dimensions (*W* = 50 mm, *L* = 50 mm) but the different thickness (d = 230 nm, d = 870 nm) were attached on the body of volunteer. The experimental results indicated the thick OECT provided better ability in electroencephalography recording (Figure 3c). However, since the electrochemical switching process of OECTs is complicated [31,32], the related fundamental mechanism behind their operation remains largely unexplored, and still more detailed work is needed to clarify the influence of geometry on device performance.

Table 1 summarizes the application of OECTs on monitoring bioelectric signals. Since OECT devices couple the functions of sensors and transistors, an OECT array consisted of several transistors exhibits an increased SNR through the signal amplification properties of transistors. Khodagholy et al. [33] fabricated an OECT array containing 17 transistors, the OECT array had a transconductance of 900 μS as well as SNR of 24.2 dB. Such high transconductance and excellent signal to noise ratio make the OECT arrays very suitable for in vivo recording of brain activity. Gu et al. [16] demonstrated the use of a 16-channel OECT array to map the real-time propagation of action potential. The transconductance and SNR were 1.1 mS and 13 dB, respectively. Except for OECT arrays, one single OECT also showed a high transconductance of 1.3 mS, the OECT device was successful in recording human electrophysiology, such as cardiac rhythm, eye movement, and brain activity [34]. Even by replacing the gate by the skin, Campana et al. [35] demonstrated the feasibility of recording an electrocardiographic signal by placing the ground contact (acting as a gate) on the chest.

### 3.2. Modification of Active Layer

Although PEDOT:PSS has proven to be an ideal material for bioelectronic applications, PEDOT:PSS film is difficult to perform functionalization after cure. Therefore, immobilization of the biorecognition element on PEDOT:PSS channel is a crucial step in the fabrication of OECT-based biosensors or biomedical devices [37,38]. Compared with the physical adhesion method, the covalent binding and cross-linking techniques are preferable due to the controllable ligand density in the matrix. After the immobilization of specific biomolecules on the activity layer, the selectivity and sensitivity of the sensor would be greatly improved. To date, various biorecognition elements including enzymes, antibodies and nucleic acids have been successfully conjugated to conducting polymers for specific applications. As shown in Table 2, after grafting biological identification molecules on PEDOT:PSS films, OECTs were successfully used to detect human influenza A virus [39], glucose [2], bacteria *E. coli* O157:H7 [6], (prostate specific antigen/α1-antichymotrypsin) PSA-ACT complex [40], et al.

**Figure 4:**
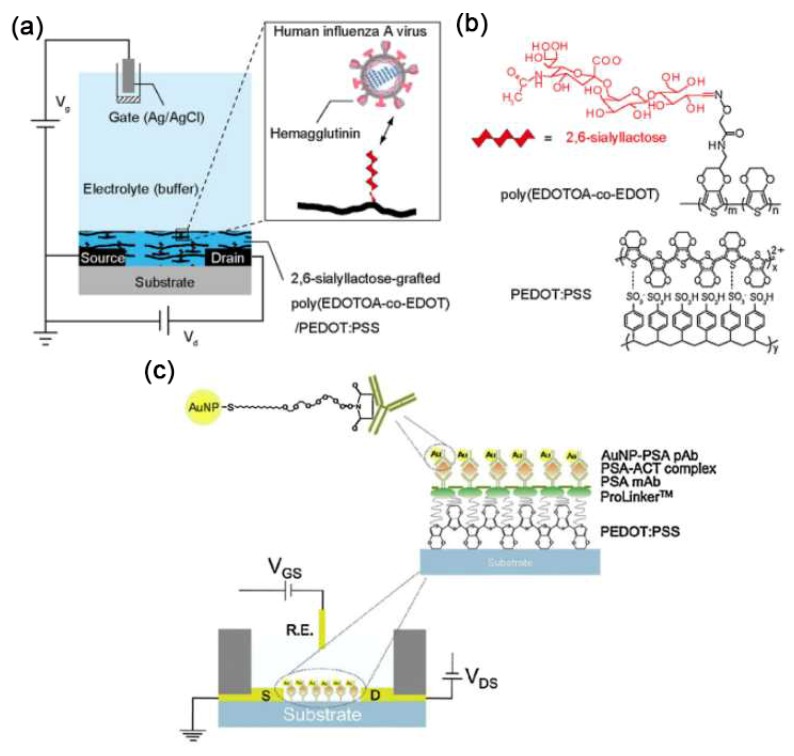
Schematic illustration of functionalization of PEDOT:PSS channel for the detection of (**a**,**b**) human influenza A virus and (**c**) prostate specific antigen. Figure reproduced with permission from references [39,40].

As shown in Figure 4a, Hai et al. [39] developed a 2,6-sialyllactose-functionalized OECT biosensor for the specific detection of human influenza A virus. Figure 4b shows the chemical structure of 2,6-sialyllactose-grafted poly(EDOTOA-co-EDOT), which was generated by the electropolymerization of 3,4-ethylenedioxythiophene (EDOT) and its adduct bearing an oxylamine group (EDOTOA) onto the PEDOT:PSS film. Because of the existence of oxylamine moiety on the EDOTOA, 2,6-sialyllactose can be easily grafted onto the composite film via a glycosylation reaction. After modification, the adsorption of negatively-charged viral nanoparticle on the channel changed the gate potential, further changing drain current. The detection limit was 0.025 hemagglutinating units (HAU), which was about two orders of magnitude lower than the conventional immunochromatographic tests. Besides, the developed OECT biosensors were comparable to other influenza virus biosensors or technologies, including mass [42], colorimetric [43], and electrical sensors [44].

Kim et al. [40] demonstrated an OECT-based immunosensor for the detection of prostate specific antigen/α1-antichymotrypsin (PSA-ACT) complex, an important biomarker for prostate hyperplasia. As shown in Figure 4c, the PDEOT:PSS film was successively modified by the AuNPs-PSA pAb, PSA-ACT complex, PSA mAb, and ProLinkerTM molecules (from the top to the bottom shown in Figure 4c). The use of AuNPs-PSA pAb can amplify the electrochemical signals between the PSA-ACT complex/PSA mAb and the OECT device, leading to a low detection limit of 1 pg/mL (much below the cut-off limit of 4 ng/mL).

He et al. [6] successfully grafted anti-*E. coli* O157:H7 antibodies on the PEDOT:PSS channel via a silane treatment, and demonstrated that OECTs can be used to detect bacteria (*E. coli* O157:H7). The detection limit of the bacteria sensor was 10^−3^ cuf mL^−1^. Besides, after functionalization of PEDOT:PSS films with enzyme glucose oxidase, OECTs showed a linear response to glucose ranging from 1.1 mM to 16.5 mM, which covered the human body blood glucose level of 3.85–8.25 mM [2].

Except for biomarkers, OECTs can be also used to selectively detect inorganic ions in water. Schmoltner et al. [45] selectively detected Na^+^ by introduction of a Na^+^-selective membrane between electrolyte and channel film. The Na^+^-selective membrane can block interfering inorganic ions and only allows Na^+^ to pass through the membrane. The OECTs showed a broad Na+ response from 10^−6^ M to 10^−1^ M. Furthermore, Sessolo et al. [41] fabricated an all-solid-state ion-selective OECT, which can detect K^+^ with a low detection limit of 15 μM. The OECT device was integrated a K^+^-selective membrane, but a hydrogel was employed as the internal electrolyte on the top of the PEDOT:PSS channel. The all-solid-state OECT is very versatile, allowing for the detection of various ions simply by introducing appropriate ion selective membrane.

## 4. Gate Surface as Sensing Interface

### 4.1. Metal Gate Electrodes

From the observation of device structure, OECTs can be divided into two separated components: bottom channel part and upper gate part. Modification of the bottom PEDOT:PSS channel often suffers from several problems [38,46]: (i) biomolecule inhomogeneous distribution limited by the low biofunctionality of PEDOT:PSS; (ii) the conductivity of PEDOT:PSS may be disrupted after biomolecule immobilization; and (iii) biomolecule may denature as a result of high-temperature hard baking. Therefore, much more effort has been devoted to the modification of the gate electrode. The main advantages can be listed as follows: on the one hand, the modification of gate electrode would not affect the performance of the channel, and a variety of modification methods can be performed without considering the deleterious effects to the PEDOT:PSS film. On the other hand, the material selection for the gate electrode is rich, and until now, various metal and semiconductor materials have been used as the gate electrodes, including Au, Pt, Ag, Ag/AgCl, ITO, TiO_2_ nanotube, and patterned PEDOT:PSS films [5,47,48,49,50,51] (see Table 3).

Tarabella et al. [52] studied the effect of the gate-electrode material selection on the OECTs response. They found that Ag gate electrode showed larger current modulation than the Pt electrode. The different responses of Ag and Pt OECTs were attributed to the Faradaic reaction between Ag and Cl^−^ in the electrolyte (Ag + Cl^−^ → AgCl + e^−^). No polarization occurred at Ag gate interface and all the gate potential was applied on the channel interface, thus increasing the channel current. Tang et al. [53] investigated the current modulation ability of OECTs using activated carbon gate electrodes. Their results indicated that activated carbon gate electrodes led to higher ON/OFF ratio (~500) when compared to PEDOT:PSS gate electrodes with the same geometric area (~15). The large current modulation was attributed to the high-specific surface area (m^2^/g) and the high specific capacitance (F/g) of the activated carbon electrode.

Owing to the separable structure of the OECT devices, the gate electrode and channel can be fabricated individually and conveniently. Zhang et al. [54] fabricated the OECT-based amino acid sensors in two steps. Firstly, the Au gate electrode was modified with L-Trp molecularly imprinted polymer (MIP) by cyclic voltammetry on an electrochemical workstation. Secondly, in order to confirm that the MIP film was successfully deposited on the surface of the Au electrode, they assessed the electrochemical properties and morphologies of the polymer films using atomic force microscopy, cyclic voltammetry and electrochemical impedance spectroscopy measurements. Thirdly, the bottom channel was individually fabricated on a glass substrate, and an unmodified Au gate electrode was used to investigate the electrical properties of the OECT devices. After confirming that the unmodified OECT devices can work normally, Zhang et al. assembled the modified gate electrode and the bottom channel to test the amino acid sensing performance. The OECT sensors modified with MIP showed highly sensitive and selective response to amino acids. As seen, the individual fabrication method is convenient for the investigation and improvement of modification technology, but will not affect the performance of bottom channel. Similarly, Gentili et al. [55] firstly immobilized the anti Interleukin-6 (IL-6) antibodies onto the surface of gate electrodes, and accessed the functionalization by cyclic voltammetry and electrochemical impedance spectroscopy. Then they tested the OECT devices with gate electrodes functionalized with anti IL-6. The detection limit of IL-6 was 220 pg/mL, within the physiological range.

Pt is commonly chosen as the gate electrodes because of its strong electro-oxidation. But the selectivity of Pt is relatively weak, and further surface modification is needed. Mark et al. [56] reported a high sensitive OECT-based epinephrine sensor with Pt gate electrode. To improve the OECT’s sensitivity, Pt gate electrodes were modified with different carbon-based materials, including single-walled carbon nanotubes (SWNTs), graphene flakes (Gr) and graphene oxide (GO), which could greatly enhance the conductivity and the electrocatalytic activity of the gate electrodes. Moreover, Pt gate electrodes could be further modified with Nafion to improve the selectivity of the devices. Drop coating was adopted to construct the composite film of Nafion and nanomaterials on the surface of the Pt gate electrodes. Because Nafion exhibits negatively charged in phosphate buffered saline (PBS) solution (pH = 7.4), and ascorbic acid and uric acid also carry negatively charged in PBS, the interference of ascorbic acid and uric acid can be neglected due to electrostatic interaction. The developed OECT devices thus showed a detection limit of 0.1 nM, which was much lower than those of the traditional electrochemical technologies.

**Figure 5:**
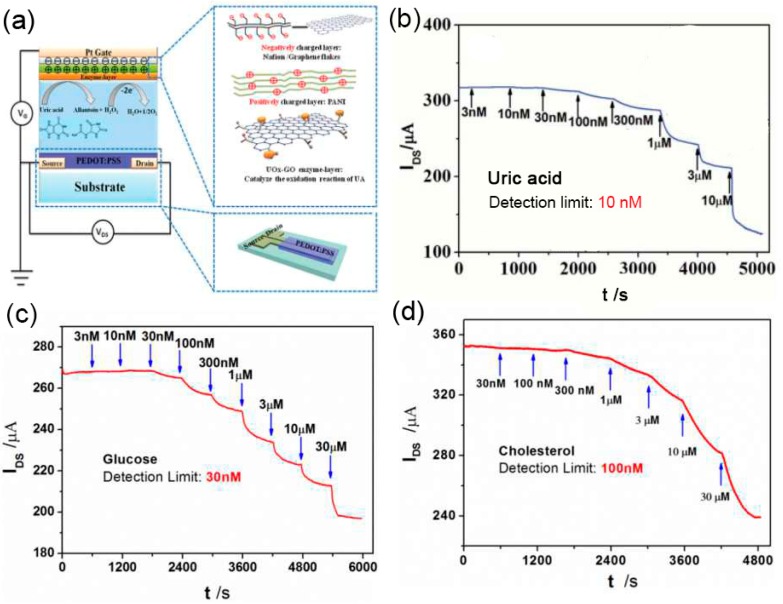
(**a**) Schematic diagram and (**b**) Current responses of a uric acid-sensitive OECT with a UOx-GO/PANI/Nafion-graphene/Pt gate. (**c**) Current responses of an OECT with GOx-GO/PANI/Nafion-graphene/Pt gate electrodes to additions of glucose. (**d**) Current responses of an OECT with ChOx-GO/PANI/Nafion-graphene/Pt gate electrodes to additions of cholesterol. Figure reproduced with permission from reference [57].

Similarly, as shown in Figure 5a, in order to improve the selectivity of OECTs to H_2_O_2_, Liao et al. [57] modified a Pt gate electrode with a PANI/Nafion-graphene bilayer film (polyaniline: PANI). In their design, the PANI film was designed to repel the positively charged molecules, such as dopamine. Nafion film was designed to repel the negative charged molecules, like ascorbic acid and uric acid. In addition, the nanochannels in bilayer film can block the big molecules like glucose, while small molecules like H_2_O_2_ can freely pass through. On the basis of this concept, as shown in Figure 5b–d, highly sensitive and selective uric acid sensors, cholesterol sensors, and glucose sensors have been constructed by immobilizing corresponding enzyme uricase (UOx), glucose oxidase (GOx), and cholesterol oxidase (ChOx) on the PANI/Nafion-graphene/Pt gate electrode, respectively. The detection limits of uric acid, glucose, and cholesterol were 10 nM, 30 nM, and 100 nM, respectively. Besides, they also compared the sensing performance with the conventional amperometric sensors, and the detection limit of uric acid is much lower than that of the conventional amperometric methods (3 μM).

PEDOT:PSS can be also used as gate electrodes in OECT devices. Till now, all PEDOT:PSS organic electrochemical transistors have been employed to detect ascorbic acid [58], lactate [12], glucose [4,59], et al. These redox active species can be electrocatalytically oxidized on the PEDOT:PSS gate, further reducing the PEDOT:PSS channel, and the change of channel current is proportional to the analyte concentration. Yaghmazadeh et al. [60] found that higher sensitivity can be obtained when the area of the gate electrode is smaller than that of the channel film. OECTs with smaller gate electrodes showed the best sensing performance for ascorbic acid [12].

**Figure 6:**
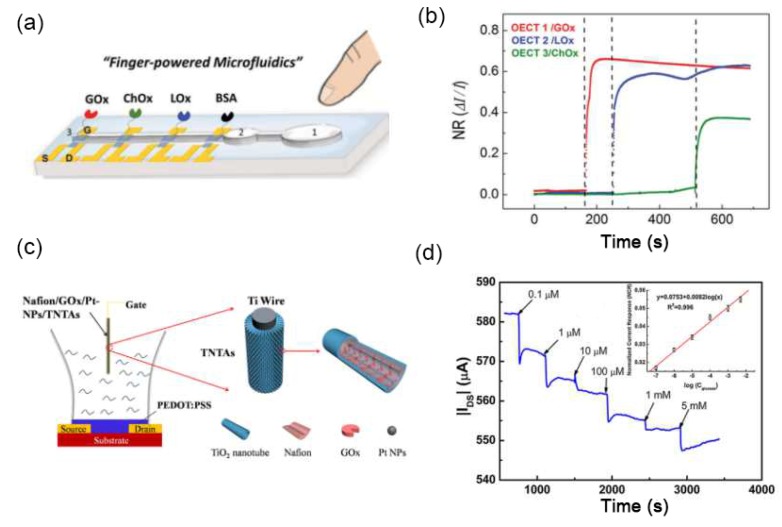
(**a**) Schematic diagram of a finger-powered PDMS microfluidic OECT platform and (**b**) the current responses to the successive additions of the three analytes. Figure reproduced with permission from reference [62]. (**c**) Schematic diagram of an OECT glucose sensor using Nafion/GOx/Pt-NPs/TNTAs as a gate electrode and (**d**) the current responses to the successive additions of glucose. Figure reproduced with permission from reference [5].

Compared with OECTs with metal gate electrodes, all-polymer OECTs offer many advantages, such as low cost, easy fabrication, and compatible with a flexible substrate. Besides, all-PEDOT:PSS OECTs facilitate the integration with microfluidic channels for point-of-care testing (POCT) applications [10,61,62,63,64]. As shown in Figure 6a, a “finger-powered” (poly(dimethylsiloxane)) PDMS microfluidic OECT platform was constructed on a glass substrate [62]. The three gate electrodes were modified with GOx, ChOx, and lactate oxidase (LOx) for the respective detection of glucose, lactate, and cholesterol. During the test, a human finger presses on the “button” to drive the liquid inside the microchannel; when the body fluid flows through the gate, the metabolites would be successfully detected (Figure 6b). OECTs can also be integrated into flexible microfluidic systems. Lin et al. [10] fabricated an OECT on a flexible PET substrate and integrated it with a PDMS microfluidic channel. The device performance was stable after being bent to both sides, and successfully detected a DNA concentration as low as 1 nM.

### 4.2. Semiconductor Gate Electrodes

Except for noble-metal gate electrodes, semiconductor gate electrodes have attracted increasing interest because of their low cost, availability, and good biocompatibility. As shown in Figure 6c,d, Liao et al. [5] demonstrated an OECT-based glucose sensor with TiO_2_ nanotube arrays (TNTAs) gate electrode. TNTAs were fabricated by electrochemical anodization, and the Pt nanoparticles were uniformly decorated on TNTAs by the ultrasound-assisted electrodeposition method. Owing to the porous structure of TNTAs, more Pt nanoparticles and enzyme GOx could be supported, which resulted in a low detection limit of 100 nM. Compared with the flat Pt-based gate electrode, the sensing performance of the OECT using the TNTAs-based gate electrode was comparable, but the cost was much lower.

Utilizing the photoelectrochemical properties of a semiconductor, a new concept of OECT, organic photoelectrochemical transistor (OPECT) has been reported by Liao et al. [50], which combined the advantage of OECT device and the photoelectrochemical method, such as low cost, high sensitivity, and low detection limit. It is well-known that TiO_2_ is an important photocatalytic material, which can decompose organic compounds under UV light irradiation. With TiO_2_ nanotube arrays as the gate electrode, Liao et al. [50] used the OPECT to detect chemical oxygen demand (COD) in wastewater. The channel current was sensitive to the photocatalytic reaction on the surface of the TiO_2_ gate, and showed a linear response to the logarithm of COD value in wastewater. Compared with the conventional photoelectrochemical (PEC) method, the OPECT COD sensor showed a detection limit as low as 0.01 mg/L, and the size has been greatly reduced because the large clumsy three-electrode system has been replaced by OPECT. Therefore, the OPECT COD sensor is highly suitable for COD onsite measurement.

**Figure 7:**
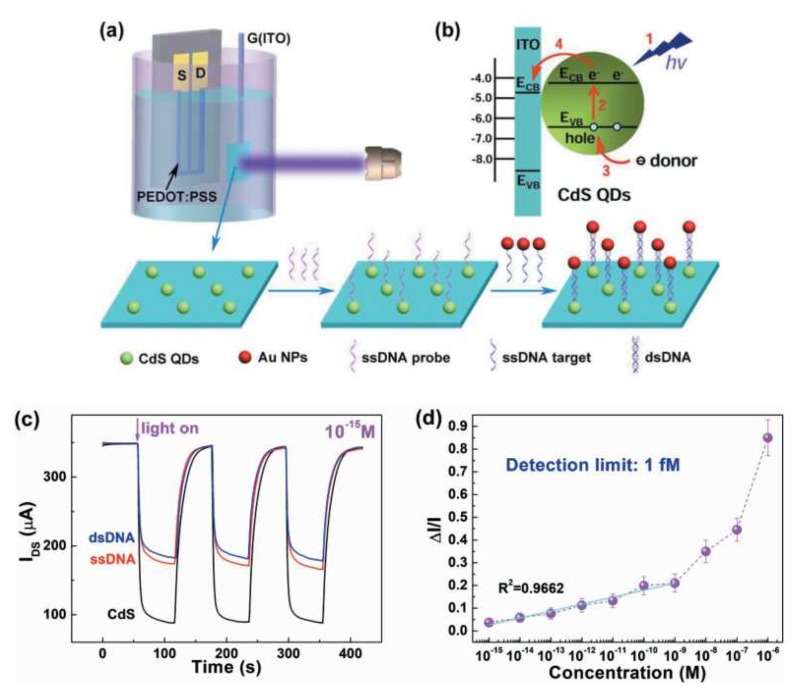
(**a**) Schematic diagram of an organic photoelectrochemical transistor (OPECT)-based biosensor for the DNA detection, and (**b**) the charge transfer between CdS quantum dots (QDs) and indium tin oxide (ITO) gate electrode under light illumination. (**c**) Channel current (*I*_ds_) versus time at several repeated light on/off cycles with the immobilization of DNA. The concentration of the ssDNA probes is 1 × 10^−15^ M. (**d**) Dependence of Δ*I*/*I* on the concentration of ssDNA targets. Figure reproduced with permission from reference [51].

Using integrated indium tin oxide (ITO) glass modified with CdS quantum dots (QDs) as gate electrode, OPECT can be also used to photoelectrochemical bioanalysis [51]. As shown in Figure 7a, under light illumination, the electron-hole pairs are generated on the surface of CdS QDs, and the electrons on the conduction band (CB) are transferred to the ITO gate electrode, which will lead to the change of the potential drop at the gate/electrolyte interface (Figure 7b). Thanks to the signal amplification of the transistor, the change of channel current response is more sensitive than conventional PEC bioanalysis. As shown in Figure 7c,d, the OPECT can detect the concentration of target DNA down to 10^−15^ M, which was one to two orders of magnitude better than that of traditional PEC bioanalysis. Moreover, compared with other reported OECT-based DNA sensors, the detection limit of OPECT-based DNA sensor was also much low, improving by nearly four orders of magnitude.

## 5. Conclusions and Outlook

OECTs are highly sensitive transducers for converting biochemical reaction into electronic signals. Two sensing interfaces, including a channel film and gate electrode, play important roles in obtaining high-performance OECT-based sensors. Accordingly, this review has summarized the development of OECT-based sensors from the standpoint of the functionalization of these interfaces. Overall, PEDOT:PSS channel film exhibits a high biocompatibility, which is beneficial to apply in bioelectric devices. High transconductance and excellent signal to noise ratio are essential to realize the recording of low-amplitude bioelectric signals. Furthermore, the effect of film thickness and width/length ratio of the PEDOT:PSS channel is discussed to gain insight into the amplification and sensitivity of OECTs. However, the fundamental mechanism behind their operation remains largely unexplored, and more detailed work is needed to clarify the influence of geometry on device performance. Moreover, OECT arrays are an excellent platform for studying physiological activities. An OECT array consisting of several transistors can easily obtain superior SNR due to the built-in amplification of transistors. Thus OECT arrays fabricated on the flexible substrates make them attractive candidates for human electrophysiology. However, the development of water-stable channel material is an urgent need in further study. Graphene-based transistors may be an attractive option for sensing applications, and the related literature has been reviewed [69,70,71].

Due to the separable structure, the gate electrodes can be fabricated and modified individually without deteriorating the electrical character of the channel. Therefore, in order to improve the sensing performance of OECTs, increasing effort has been devoted to the surface modification of gate electrodes. For example, H_2_O_2_ is a main product of enzymatic reaction, so most enzyme biosensors are based on the electrochemical detection of H_2_O_2_. It is known that Pt nanoparticles are an excellent electrocatalyst for H_2_O_2_, and carbon nanotubes or graphene possess good conductivity and capacity for biomolecule immobilization. Therefore, utilizing the signal amplification of nanomaterials, modification of gate electrodes with Pt nanoparticles and/or carbon-based material can increase the sensitivity of OECT-based enzymatic sensors. With further modification with Nafion films, the selectivity of the devices would be greatly improved [8]. Based on the same principle, OECTs will find wide applications by immobilizing suitable enzymes on the gate surface as long as H_2_O_2_ can be generated by corresponding enzymatic reaction.

Recently, combination with the photoelectrochemical technology, a new concept of OPECT has been proposed, which brings a new vista to the traditional photoelectrochemical sensors. As a new type of biosensor, the research on OPECT devices is still limited, because OPECT is an interdisciplinary field which covers the photoelectrochemistry, semiconductor device physics and materials science. Photosensitive gate electrode is the core element of OPECT devices. Following the research experience of traditional photoelectrochemical sensors, the development and improvement of novel photosensitive semiconductor, photo-generated carrier transfer efficiency, and the extension of the optical absorption spectra are key points to build high-efficiency OPECT-based sensing platform.

The main drawback of OECT devices is the selectivity. Although an interface modification method can greatly improve its selectivity, and great achievements have been obtained, the method is still complicated and time-consuming. Recently, a potentiodynamic approach has been demonstrated to increase the selectivity of dopamine in the presence of interfering compounds (ascorbic acid and uric acid) [9,72]. By varying the gate voltage and the scan rate, the electro-oxidation peaks of the three different analytes (ascorbic acid, uric acid and dopamine) were separated, and the peak currents showed a linear correlation with the analyte concentrations. Except for the potentiodynamic approach, the combination electrochemical impedance technology with OECTs can also address the selectivity of OECTs [73].

In summary, OECTs are promising devices for applications in in vitro and in vivo measurements. Introduction of specific chemical functionalities at the sensing interfaces is a crucial role in realizing high-performance OECT-based sensors, and understanding the mechanisms behind the observed phenomena is still challenging. Systematic studies in terms of experiments and simulation are of great importance in future work.

## Figures and Tables

**Table 1:** Application of OECTs with high transconductance and excellent signal to noise ratio in monitoring bioelectric signals.

Channel (Gate)	Target	Performance	Parameters	Ref.
PEDOT:PSS (Ag/AgCl)	Electrophysiological activity	*g*_m_ = 12 mS	16 transistors *L* = 200 μm, *W* = 200 μm	[15]
PEDOT:PSS (Ag/AgCl)	Cardiac action potentials	*g*_m_ = 1.1 mS SNR = 13 dB	15 transistors *L* = 20 μm, *W* = 70 μm	[16]
PEDOT:PSS (Ag/AgCl)	Cardiac action potentials	*g*_m_ = 100 mS SNR = 7 dB	27 transistors *L* = 24 μm, *W* = 38 μm	[17]
PEDOT:PSS (Steelless)	Brain activities	*g*_m_ = 900 μS SNR = 24.2 dB	17 transistors *L* = 6 μm, *W* = 15 μm	[33]
PEDOT:PSS (Ag/AgCl)	Cardiac rhythm Eye movement Brain activity	*g*_m_ = 1.3 mS	1 transistor *L* = 100 μm, *W* = 100 μm	[34]
PEDOT:PSS (Ag/Pt wire)	Electrocardiographic recording	*g*_m_ = 3.2 mS	1 transistor *L* = 30 μm, *W* = 1000 μm	[35]
PEDOT:PSS (Ag/AgCl)	Action potentials fromcardiomyocyte cells	*g*_m_ = 2.5 mS SNR = 4 dB	4 transistors *L* = 30 μm, *W* = 40 μm	[36]

**Table 2:** Functionalization of OECT channel surface for bioelectric applications.

Channel (Gate)	Target	Channel Functionalization	Performance	Ref.
PEDOT:PSS (N^+^-Si)	Glucose	GOx	Linear range 1.1–16.5 mM	[2]
PEDOT:PSS (Ag/AgCl)	*E. coli* O157:H	Anti-*E. coli* O157:H7 antibodies	Detection limit 10^−^^3^ cuf mL^−^^1^	[6]
PEDOT:PSS (Ag/AgCl)	Human influenza virus	Trisaccharides	Detection limit 0.025 HAU	[39]
PEDOT:PSS (Ag/AgCl)	PSA-ACT complex	Au NPs + PSA pAb	Detection limit 1 pg/mL	[40]
PEDOT:PSS (Ag/AgCl)	K^+^	K^+^ ion-selective membrane	Linear range 10^−^^4^–10^−^^1^ M	[41]

**Table 3:** Functionalization of OECT gate surface for bioelectric applications.

Gate (Channel)	Target	Gate Electrode Functionalization	Performance	Ref.
Au (PEDOT:PSS)	Amino acid	Molecularly imprinted polymer	Linear range 300–10μM Sensitivity 3.19 μA/μM Detection limit 2 nM	[54]
Au (PEDOT:PSS)	Interleukin-6	IL-6 antibodies	Detection limit 2 ng/mL	[55]
Au (PEDOT:PSS)	Gallic acid	PDDA + carbon nanomaterials	Linear range 1–10 μM Detection limit 10 nM	[65]
Au (PEDOT:PSS)	Glucose Lactate	GOx/LOx + Pt NPs	Detection limit 0.1 μM glucose 1 μM lactate	[66]
Pt (PEDOT:PSS)	Epinephrine	Nafion + SWNTs	Detection limit 0.1 nM	[56]
Pt (PEDOT:PSS)	Uric acid	UOx-GO + PANI + Nafion + graphene	Detection limit 10 nM	[57]
Pt (PEDOT:PSS)	Dopamine	Nafion + graphene	Detection limit 5 nM	[8]
Pt (PEDOT:PSS)	Glucose	CHIT/GOx/Pt-NPs	Detection limit 5 nM	[3]
PEDOT:PSS (PEDOT:PSS)	Glucose Lactate Cholesterol	GOx LOx ChOx	Linear range 0.02–1 mM glucose 0.1–2 mM lactate 0.01–0.7 mM cholesterol Detection limit 10 μM glucose 50 μM lactate 10 μM cholesterol	[62]
PEDOT:PSS (PEDOT:PSS)	Ascorbic acid	-	Detection limit 80 μM	[58]
PEDOT:PSS (PEDOT:PSS)	Lactate	Lox + CHIT + Fc	Linear range 30–300 μM Detection limit 10 μM	[12]
PEDOT:PSS (PEDOT:PSS)	Glucose	GOx	Linear range 10^−^^7^–10^−^^2^ M	[67]
Ag/AgCl (PEDOT:PSS)	Bacteria	-	Detection limit 10^−^^3^ cuf mL^−^^1^	[6]
Ag/AgCl (PEDOT:PSS)	K^+^ Ca^2+^ Al^3+^	-	Linear range 10^−^^3^–10^−^^1^ M	[1]
ITO (PEDOT:PSS)	DNA	CdS QDs + ssDNA probe (420 nm light illumination)	Linear range 10^−^^15^–10^−^^9^ M Detection limit 10^−^^15^ M	[51]
glass carbon electrode (PEDOT:PSS)	Sialic acid	Poly (3-aminophenylboronic acid)	Linear range 8 μM–2 mM Detection limit 8 μM	[68]
TiO_2_ nanotube arrays (PEDOT:PSS)	Glucose	Nafion/GOx/Pt-NPs	Linear range 100 nM–5 mM Detection limit 100 nM	[5]
TiO_2_ nanotube arrays (PEDOT:PSS)	Chemical oxygen demand	UV-LED as excited light source	Detection limit 0.01 mg/L	[50]
